# Environmental enrichment reverses Aβ pathology during pregnancy in a mouse model of Alzheimer’s disease

**DOI:** 10.1186/s40478-018-0549-6

**Published:** 2018-05-31

**Authors:** Stephanie Ziegler-Waldkirch, Karin Marksteiner, Johannes Stoll, Paolo d´Errico, Marina Friesen, Denise Eiler, Lea Neudel, Verena Sturn, Isabel Opper, Moumita Datta, Marco Prinz, Melanie Meyer-Luehmann

**Affiliations:** 1 Department of Neurology, Medical Center – University of Freiburg, Freiburg, Germany; 2 Faculty of Medicine, University of Freiburg, Freiburg, Germany; 3 Faculty of Biology, University of Freiburg, Freiburg, Germany; 4 Institute of Neuropathology, Medical Center – University of Freiburg, Freiburg, Germany; 5 BIOSS Centre for Biological Signalling Studies, University of Freiburg, Freiburg, Germany; 6 Neurocenter, University of Freiburg, Breisacher Str.64, 79106 Freiburg, Germany

**Keywords:** Alzheimer’s disease, Pregnancy, Amyloid-β plaques, Adult neurogenesis, Estrogens, Progesterone, Environmental enrichment

## Abstract

Several studies suggest that women have a higher risk to develop Alzheimer’s disease (AD) than men. In particular, the number of pregnancies was shown to be a risk factor for AD and women with several pregnancies on average had an earlier onset of the disease, thus making childbearing a risk factor. However, the impact of being pregnant on Aβ plaque pathology and adult neurogenesis still remains elusive. Postmortem analysis revealed that pregnant 5xFAD transgenic mice had significantly more Aβ plaques in the hippocampus from G10 onwards and that the number of Ki67 and DCX positive cells dramatically decreased during the postpartum period. Furthermore, 5 months old 5xFAD transgenic mice that also nursed their offsprings for 4 weeks had a similar Aβ plaque load than merely pregnant mice, indicating that pregnancy alone is sufficient to elevate Aβ plaque levels. Interestingly, housing in an enriched environment reduced the Aβ plaque load and vivified neurogenesis. Our results suggest that pregnancy alters Aβ plaque deposition in 5xFAD transgenic mice and diminishes the generation of newborn neurons. We conclude that pregnancy alone is sufficient to induce this phenotype that can be reversed upon environmental enrichment.

## Introduction

Alzheimer’s disease (AD) is the most common type of dementia that is characterized by a decline in memory. The majority of AD cases are sporadic [9] and the greatest non-genetic risk factors for the development of AD are age and gender. The incidence of the disease is higher in women than in men, which was previously attributed to the higher longevity of women versus men. Several epidemiological studies suggest however that there may be other factors contributing to the disease beyond longevity. In particular, women with multiple pregnancies had an earlier onset of AD [6, 7, 31] and nulliparous women had significantly less age related cognitive decline [25], while the number of children had no effect on the risk of developing AD [31]. Together, these studies indicate reproductive experience being a risk factor for AD, but the underlying mechanism for this higher risk of AD in women is still largely unknown.

In agreement with those aforementioned studies, gender-dependent elevated Aβ plaque formation has been observed in several APP transgenic mouse models. Female APP transgenic mice that exhibit massive cerebral amyloid deposition while aging developed more Aβ pathology with an earlier onset than their male siblings [1, 5, 14, 35, 39]. Additionally, fertility experience of those female APP transgenic mice altered Aβ plaque pathology, resulting in even higher Aβ plaque numbers and larger plaque sizes when compared to non-breeders [8]. Furthermore, these neuropathological changes were associated with spatial memory impairment and the down-regulation of synaptophysin levels in APP transgenic breeders.

Adult neurogenesis is a lifelong process in the healthy adult mammalian brain, where functional neurons are generated from adult neural stem cells and adult neural precursor cells. Emerging evidence has indicated that altered neurogenesis in the adult hippocampus represents a critical event in the course of AD and that cognitive deficits, learning difficulties and memory loss in AD patients may be due to impaired adult neurogenesis [10, 15, 20, 21]. Intriguingly, environmental enrichment and voluntary exercise were shown to increase the number of newborn granule cells in the dentate gyrus [18, 19, 26, 37, 38] and to reduce Aβ plaque formation in APP transgenic mice [16, 22]. Despite the immense interest in the therapeutic potential of environmental enrichment during pregnancy, the impact on the mother however still remains elusive since most studies focus on the offspring [3, 13].

Therefore, we aimed to investigate neuropathological changes such as Aβ plaque load and adult neurogenesis during the course of pregnancy at different gestation days. Our results demonstrate that pregnant 5xFAD transgenic mice had a significantly higher Aβ plaque load in different brain regions such as hippocampus, cortex and the olfactory bulb. Interestingly, 5 month old 5xFAD transgenic mice that also nursed their offspring for 4 weeks had an Aβ plaque load similar to merely pregnant mice, indicating that pregnancy alone is sufficient to elevate Aβ plaque levels. Furthermore, neurogenesis was dramatically decreased in both groups during the postpartum period compared to 5xFAD mice. Exposure of pregnant 5xFAD mice to an enriched environment reduced Aβ deposits and stimulated the generation of newborn neurons, thus underlining the beneficial effects of exercise and enrichment during pregnancy.

## Material and methods

### Animals

We used heterozygous 5xFAD transgenic mice co-expressing human APP ^K670N/M671L (Sw) + I716V (Fl) + V717I(Lo)^ and PS1^M146L + L286V^ under the control of the neuron-specific Thy-1 promoter [27]. We backcrossed 5xFAD mice with C57BL/6 mice to generate heterozygous 5xFAD mice and non-transgenic littermates on the same genetic background. Only female mice were used in the present study. Animals were group-housed under specific pathogen-free conditions. Mice were kept under a 12-h light, 12-h dark cycle with food and water ad libitum. All animal experiments were carried out in accordance with the policies of the state of Baden-Württemberg under license number X14/02A and X17/01A.

Female mice were mated at the age of 12 weeks. The matings were initiated in the afternoon and the mice were separated after a positive plug check on the following morning. The day of vaginal plaque formation was estimated as gestation day 1 (G1). In order to analyze female mice at different stages of pregnancy, they were sacrificed on G5, G10 and G18. Non- pregnant 5xFAD female mice of the same age and from the same litters were used as control groups. To study the lactation period, female mice were mated at the age of 12 weeks and were divided into three groups. One group delivered and lactated the offspring for 4 weeks. One group was separated from their litter directly after the delivery and the third group was not pregnant and did not lactate. All mice were analyzed at postpartum day 20 (P20). To study how multiple pregnancies might affect Aβ pathology, 10 months old female mice were analyzed. Two groups of female mice, nulliparous and multiparous (4 times pregnant and lactating) were used.

### Environmental enrichment

5xFAD female mice were mated at the age of 12 weeks. One group of mice was housed in standard cages and another group of mice in an enriched environment until G18 or P20. The environmental enrichment consisted of larger cages (40 × 60 cm) that contained 1 running wheel, tunnel systems, small plastic houses and extra nesting material. The animals had free access to the running wheel. Mice were housed in groups of 5. All mice were sacrificed at the end of the experiment at G18 or P20.

### Histology

Mice were deeply anesthetized with a mixture of ketamine (300 mg per kg) and xylazine (20 mg per kg) before they were transcardially perfused with 20 ml of ice-cold PBS followed by 20 ml of ice-cold 4% paraformaldehyde in PBS. Brains were isolated and postfixed in 4% PFA for 24 h, followed by incubation in 30% sucrose (in PBS, pH 7.5) for a further 48 h. Frozen brains were cut into 25 μm thick coronal sections on a sliding microtome (SM2000R, Leica Biosystems, Wetzlar, Germany) and collected in PBS. Immunohistochemistry was performed on free floating sections using the following antibodies diluted in PBS containing 5% normal goat serum and 0.5% Triton X-100: anti-Aβ 3552 (rabbit polyclonal antibody specific for Aβ 1–40 [40] (kindly provided by E. Kremmer, Ludwig Maximilians University, Munich, Germany); diluted 1:3000), anti-doublecortin (rabbit, DCX; 1:5000, abcam, ab18723), anti-Ki67 (rabbit, 1:500, abcam, ab15580). Appropriate secondary antibodies conjugated to Alexa 488 or 555 (1:1500) were used. Dense-core plaques were stained with Thiazinred (Sigma, T3272). Staining was done according to standard protocols. In brief, sections were washed 3 times in PBS and incubated in Thiazinred (2 μM solution in PBS) for 5 min at RT followed by 3 × 5 min washes with PBS.

### Assessment of Aβ and cell analysis

Fluorescence images of brain slices were taken using a Zeiss fluorescent microscope (Axio Imager M2M). For analysis every tenth brain section of one hemisphere was used. The total Aβ load was determined by counting the Aβ-positive stained plaques using the imaging software ImageJ (National Institutes of Health freeware). Ten sections per animal were analyzed for the hippocampus and cortex and 8 sections per animal for the olfactory bulb, in total 4–6 animals per group were investigated. Aβ plaque load is presented in % and normalized to the non-pregnant control group (set to 100%).

The cell number was quantified by counting the number of positively labeled cells in the animal’s whole dentate gyrus. Four to six animals per group and 10 sections per animal were analyzed. Cell counting was done in the dentate gyrus and its area was measured with the ImageJ software. The area of the gyrus was defined according to the mouse brain atlas [29]. Cell numbers are presented as the quantity of positively labeled cells per mm^2^. All analyses were conducted in a blinded manner.

### Western blot analysis

Total protein from hippocampus was extracted in RIPA buffer (50 mM HEPES pH 7.5, 150 mM NaCl, 1 mM EDTA, 10% Glycerin, 1% Triton X-100, 10 mM Na_4_O_7_P_2_, Protease Inhibitor). Protein concentration was determined by Pierce BCA Protein Assay Kit (ThermoFisher). Samples were separated by 4–12% NuPAGE Bis-tris mini gels using NuPAGE LDS sample buffer, NuPAGE sample reducing agent and NuPAGE MES SDS running buffer (Invitrogen). Proteins were transferred on PVDF membranes (Biorad) and visualized using Clarity Western ECL Substrate (Biorad). Antibodies against APP and CTFs (rabbit polyclonal antibody against the APP C-terminus, 6687, 1:1000) [34], anti-Aβ (mouse, 1:3000, Covance, 6E10), anti-insulin degrading enzyme (anti-IDE) (rabbit, 1:5000, abcam, ab109538), anti-neprilysin 1:2500 (rabbit, 1:2500, abcam, ab79423) and anti-β-actin-HRP (mouse, 1:5000, abcam, ab20272) were used.

### ELISA

For the measurement of estrogen and progesterone levels, blood samples were taken from the ventricle before the perfusion of the mice at G5, G10 or G18. Blood was centrifuged immediately for 10 min at 1800 g. The supernatant was frozen at − 20 °C until analysis. Serum samples were prepared and analyzed by Mouse/Rat Estradiol ELISA kit (Calbiotech, catalog no. ES180S-100) or Progesterone ELISA Kit (Enzo, Enzo Life Science) according to the manufacturer’s protocols. Samples were measured in duplicates and the ELISA was conducted in two to three independent experiments.

For the quantification of Aβ40 and 42 species in the soluble and insoluble hippocampal extracts, hippocampal tissue was homogenized (10% *w*/*v*) in PBS + protease inhibitor and sequentially extracted with PBS (soluble fraction), PBS + 0.1% TritonX100 (membrane bound fraction) and finally with 8 M guanidine hydrochloride solution. Protein concentration in each fraction was measured with Bradford reagent (Roth) and ELISA was performed using Human Aβ42 ultrasensitive ELISA kit (Life Technologies, catalog no. KHB3544) and Human Aβ40 ELISA kit (Life Technologies, catalog no. KHB3481), according to the manufacturer’s protocol.

### Statistical analysis

GraphPad Prism 6 (GraphPad Software, Inc) was used for statistical analysis. All data sets were tested for normality with the D’Agostino-Pearson omnibus K2 normality test with a significance level set to *P* = 0.05 before carrying out the appropriate parametric or nonparametric statistical comparison test. The Mann-Whitney test or Kruskal-Wallis test followed by Dunn’s posthoc test were applied. Reported values are means ± S.D. Significance level α was set to 0.05. *P < 0.05; **P < 0.01; ***P < 0.001.

## Results

### Pregnancy-related alteration of Aβ plaque pathology in female 5xFAD mice

**Fig. 1:**
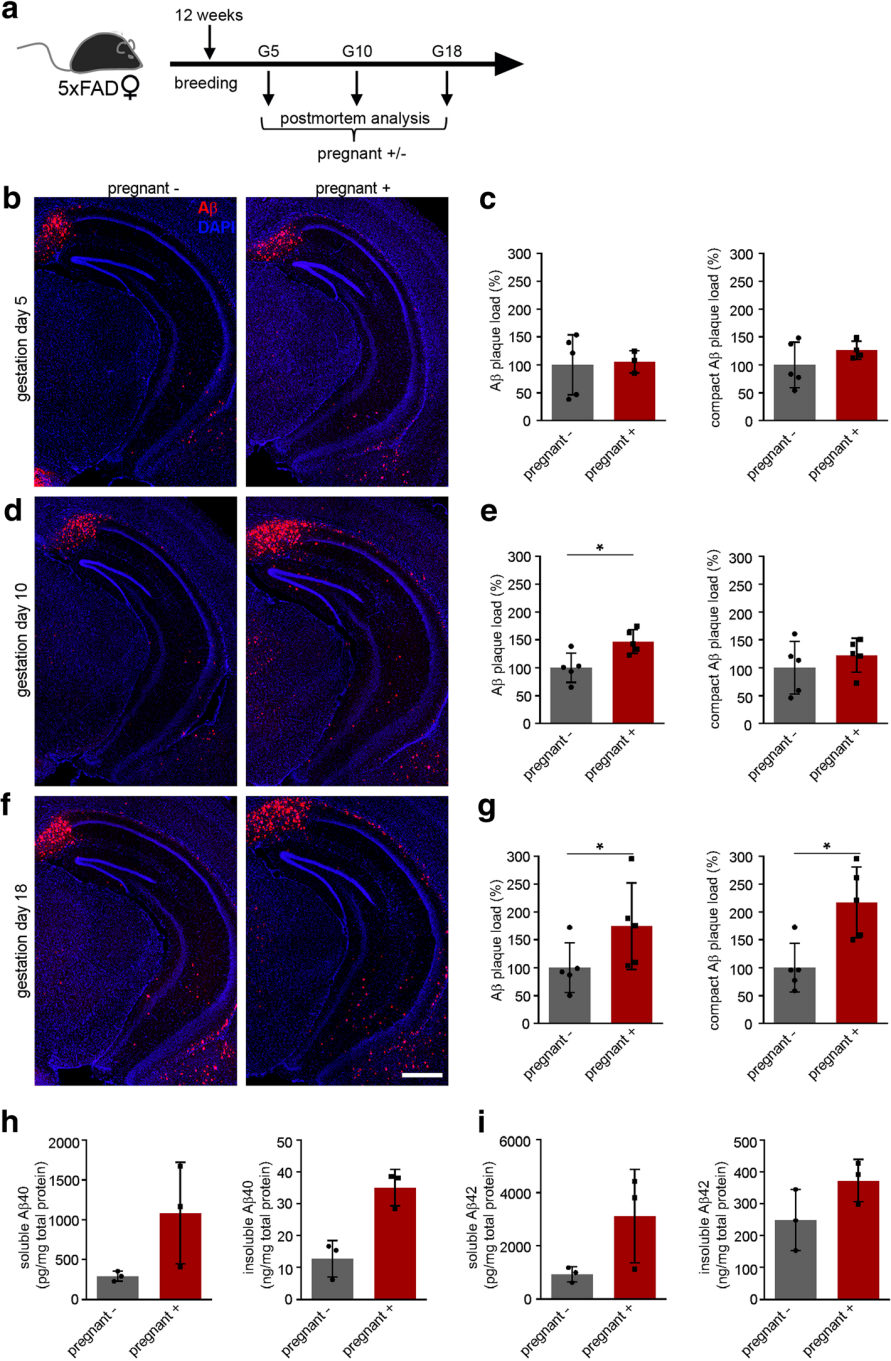
Aβ plaque load increases during pregnancy from gestation day 5 to gestation day 18 in 5xFAD female mice. **a** Schematic overview of the experimental setup. Twelve weeks old 5xFAD female mice were breed and either sacrificed at gestation day 5 (G5), G10 or G18. **b** Fluorescence microscopy of Aβ (red) and DAPI (blue). Shown are representative images of hippocampi from female 5xFAD mice not pregnant or pregnant at G5. **c** Quantification of Aβ plaque load (left) and compact Aβ plaque load (right) of not pregnant and pregnant 5xFAD mice at G5 showed no differences between the groups. **d** Fluorescence microscopy of Aβ (red) and DAPI (blue). Shown are representative images of hippocampi from female 5xFAD mice not pregnant or pregnant at G10. **e** Aβ plaque load (left) is significantly increased in pregnant 5xFAD female mice compared to not pregnant mice at G10. Mann-Whitney test, *P = 0.03. **f** Fluorescence microscopy of Aβ (red) and DAPI (blue). Shown are representative images of hippocampi from female 5xFAD mice not pregnant or pregnant at G18. **g** The number of Aβ plaques exhibited significant increase in pregnant 5xFAD mice compared to not pregnant 5xFAD mice at G18. Mann-Whitney test, *P = 0.03. **h** Sandwich immunoassay for Aβ40 soluble and insoluble fractions of hippocampal brain extracts from non-pregnant and pregnant female 5xFAD mice at G18 (*n* = 3). **i** Sandwich immunoassay for Aβ42 soluble and insoluble fractions of hippocampal brain extracts from non-pregnant and pregnant female 5xFAD mice at G18 (*n* = 3). Data are presented as mean ± S.D. Each symbol represents data from one mouse, with four to five mice per group. Scale bar represents 500 μm. G = gestation day

**Fig. 2:**
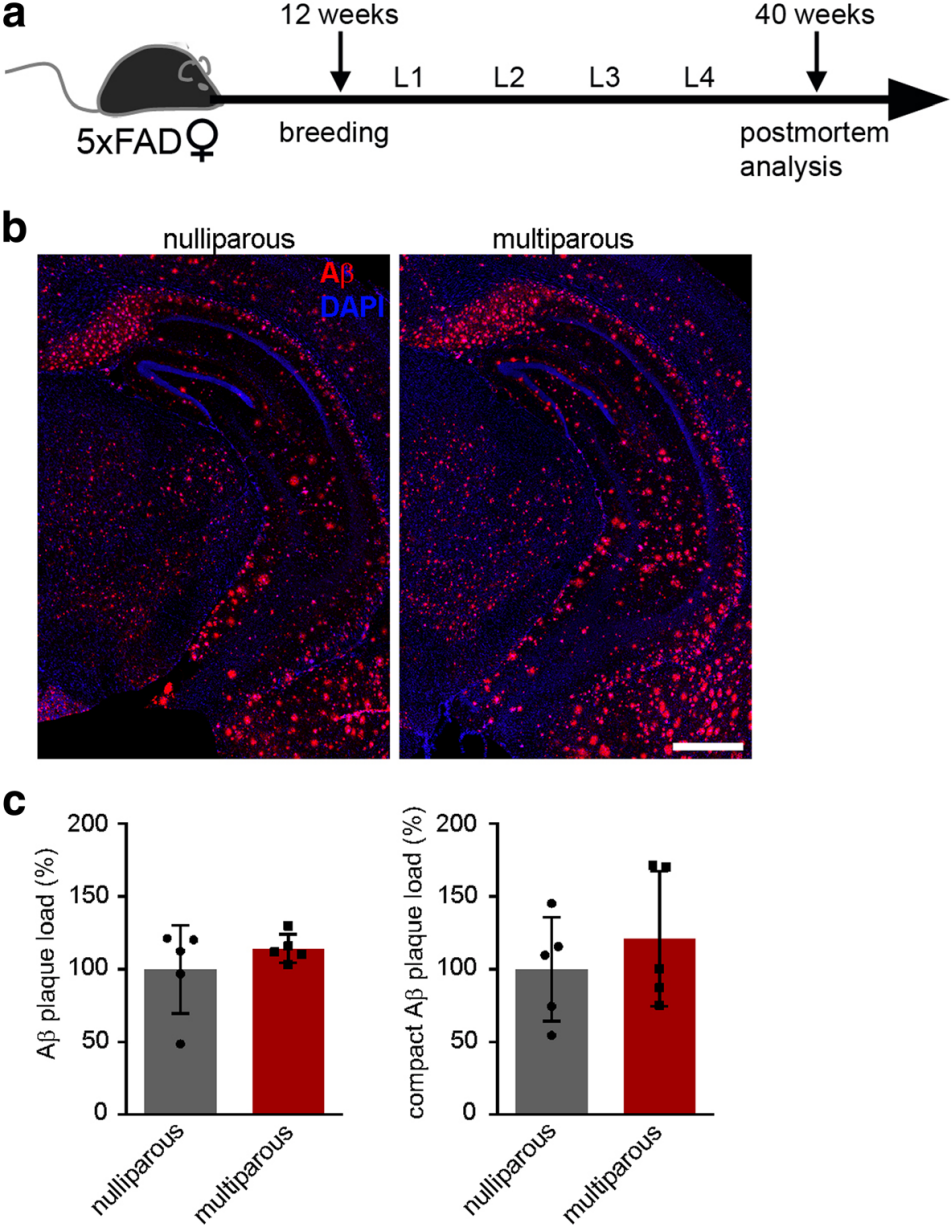
Unaltered Aβ plaque load in 10 months old nulliparous and multiparous 5xFAD female mice. **a** Schematic overview of the experimental setup. Twelve weeks old 5xFAD female mice were bred and sacrificed at the age of 10 months. Females had either no litter or 4 litters. **b** Fluorescence microscopy of Aβ (red) and DAPI (blue). Shown are representative images of hippocampi from nulliparous and multiparous female 5xFAD mice. **c** Quantification of Aβ plaque load (left) and compact Aβ plaque load (right) of nulliparous and multiparous 5xFAD mice showed no differences between the groups. Data are presented as mean ± S.D. Each symbol represents data from one mouse, with five mice per group. Scale bar represents 500 μm. L = litter

To investigate if pregnancy induces pathological alterations, we first determined the Aβ plaque load in 5xFAD transgenic female mice at different stages of pregnancy/gestation days and compared them to their age-machted non-pregnant littermates. 5xFAD transgenic mice were mated at the age of 12 weeks and were analyzed on gestation day 5, 10 and 18 (G5, 10, 18) (Fig. 1a). At G5, there was no difference between the Aβ plaque load of pregnant mice and non-pregnant littermates (Fig. 1b and c). While the total number of Aβ plaques was already increased in pregnant mice at G10, particularly in the subiculum, the number of thiazin red positive compact Aβ plaques did not differ (Fig. 1d and e). At G18, both, the number of total and of compact Aβ plaques significantly increased in pregnant 5xFAD mice when compared to controls (non-breeders) at the same age (Fig. 1f and g). In accordance with this higher plaque load in pregnant 5xFAD mice, we found that soluble and insoluble levels of Aβ40 and Aβ42 were increased but did not reach statistical significance in pregnant mice at G18 (Fig. 1h and i). To further determine if multiple pregnancies augment this effect, we next analyzed 40 weeks old female 5xFAD mice that had been pregnant 4 times (multiparous) and compared them with nulliparous mice of the same age (Fig. 2a). Although many plaques were detected in the hippocampi of nulliparous and multiparous 5xFAD transgenic mice (Fig. 2b), Aβ plaque load did not differ between both groups (Fig. 2c) most likely due to an Aβ overload.

**Fig. 3:**
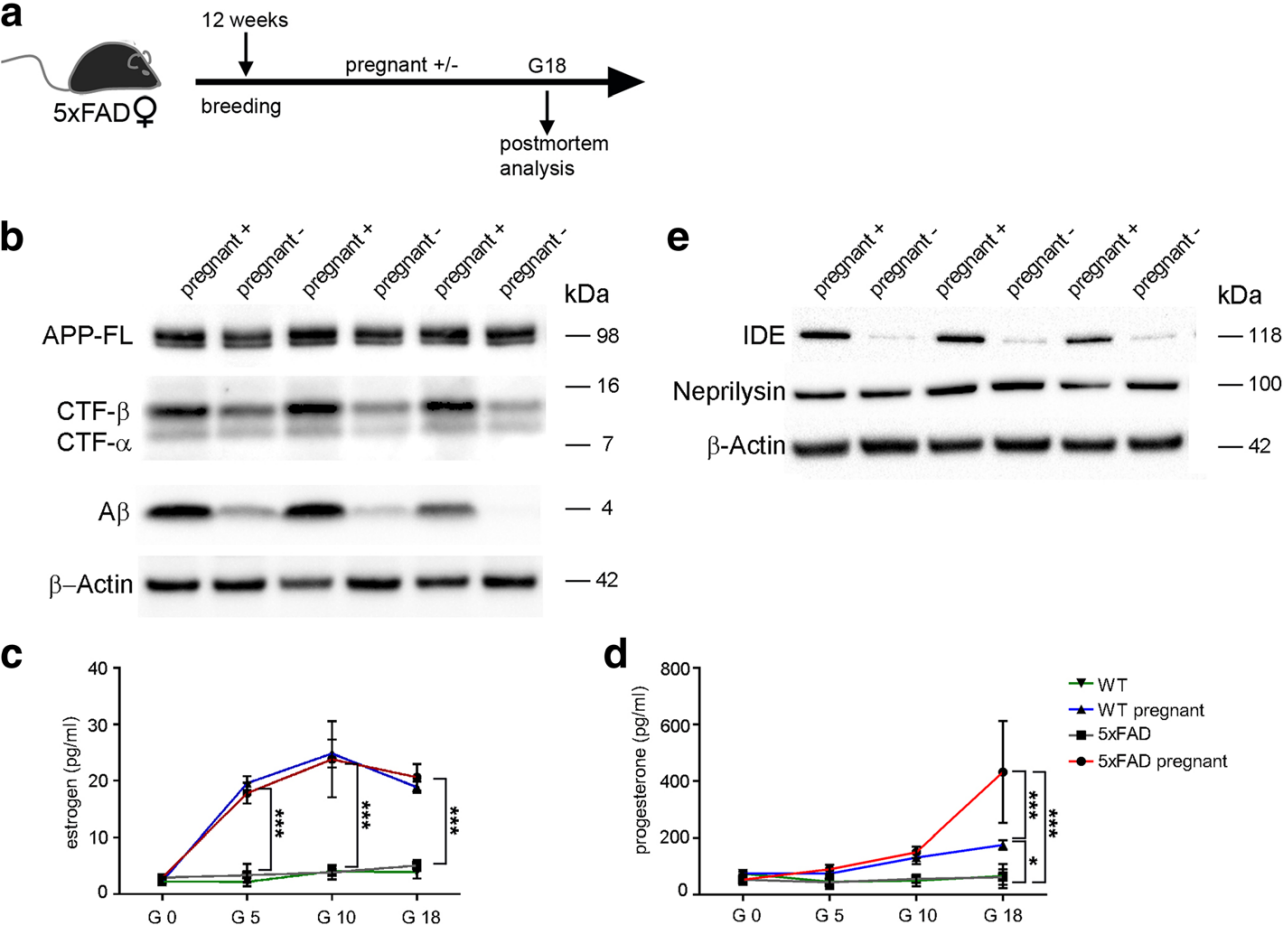
APP processing is altered in pregnant 5xFAD mice during pregnancy. **a** Schematic overview of the experimental setup. Twelve weeks old 5xFAD female mice were bred and sacrificed at gestation day 18. **b** Immunoblot analysis of hippocampal brain homogenates from pregnant and non-pregnant 5xFAD mice at G18. Immunoblots were probed with antibodies that recognize human full length APP, CTF-β and CTFα (6687) and Aβ (6E10). β-Actin was used as loading control. Pregnant 5xFAD mice showed higher levels of CTF-β and Aβ while APP levels were unaltered. **c** Serum estrogen levels of 5xFAD and WT female mice were measured by ELISA at G0, G5, G10 and G18. Estrogen levels increased during pregnancy in 5xFAD and WT mice (***P < 0.0001, ***P < 0.0001; ***P < 0.0001). **d** Serum progesterone levels of female 5xFAD and WT mice were measured by ELISA at G0, G5, G10 and G18. Progesterone levels increased significantly during pregnancy in 5xFAD and WT mice (*P = 0.03; ***P < 0.0001; ***P < 0.0001). **e** Levels of IDE and neprilysin were detected by western blotting in pregnant and non-pregnant 5xFAD mice. β-Actin was used as loading control. Data are presented as mean ± S.D. Three mice per group were used. G = gestation day, IDE = insulin-degrading-enzyme

In order to elucidate whether higher Aβ plaque load and Aβ levels in pregnant mice (G18) (Fig. 3a) result from either increased Aβ production or decreased Aβ degradation, we performed Western blot analyses. While full length APP levels were similar, there was a strong increase in monomeric Aβ and APP C-terminal fragments (in particular CTF-β) in pregnant mice, indicating that pregnancy affects the processing of APP and augments Aβ production (Fig. 3b).

Next, we collected blood samples to determine serum estrogen and progesterone levels of non-pregnant and pregnant 5xFAD female mice at G0, G5, G10 and G18. ELISA measurements revealed significant increases in estrogen levels both in pregnant WT and 5xFAD female mice from G5 onwards (Fig. 3c). Serum progesterone levels were enhanced at G10 in pregnant WT and 5xFAD mice. At G18, pregnant WT mice exhibited significantly higher progesterone levels when compared to non-pregnant littermates. Moreover, pregnant 5xFAD mice showed dramatically increased progesterone levels that even exceeded those of pregnant WT mice at G18 (Fig. 3d).

To rule out any effect of pregnancy on the clearance or degradation of Aβ, we determined the protein levels of the enzymes involved in Aβ degradation, neprilysin and IDE, which revealed mixed results. While neprilysin levels were not affected by pregnancy, we found increased IDE levels as measured by Western blotting in the hippocampus of pregnant mice (Fig. 3e), probably due to higher progesterone levels (Fig. 3d) that were shown to significantly increase IDE in vivo in rats [17].

Together, these data suggest that pregnancy modulates Aβ plaque load by increasing the generation of Aβ.

### Pregnancy and lactation impair Aβ plaque load in several brain regions of 5xFAD mice

**Fig. 4:**
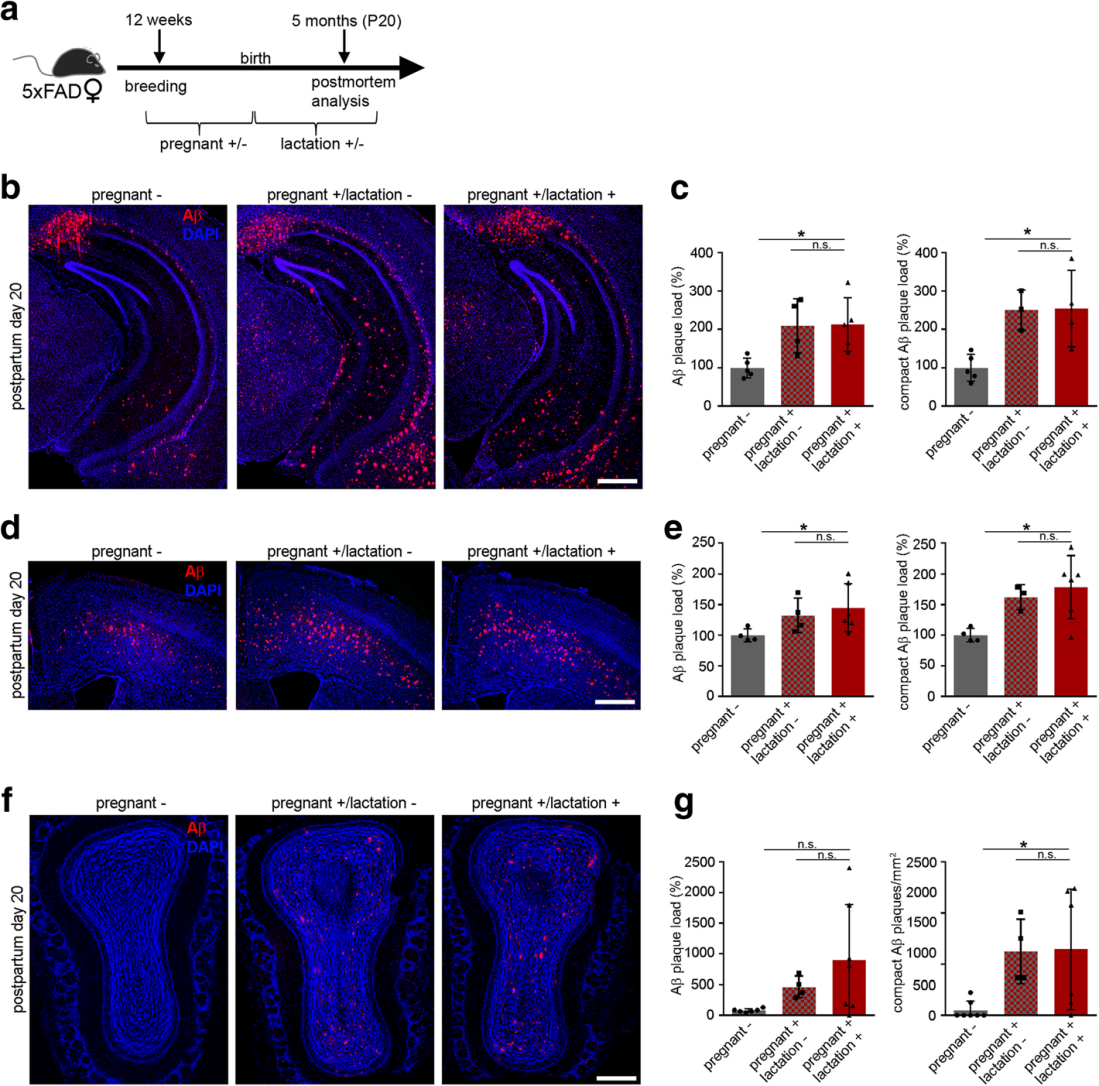
Pregnancy and not lactation increases Aβ plaque pathology in different brain regions of 5xFAD female mice. **a** Schematic overview of the experimental setup. Twelve weeks old 5xFAD female mice were mated and sacrificed at the age of 5 months (postpartum day 20 (P20)) after the pregnancy and lactation period. One group of mice was separated from the litter directly after the delivery and analyzed 4 weeks later at P20. **b** Fluorescence microscopy of Aβ (red) and DAPI (blue). Shown are representative images of hippocampi from female 5xFAD mice either non-pregnant, pregnant but not lactating or pregnant and lactating at P20. **c** Quantification of Aβ plaque load exhibited significant increases in pregnant and lactating 5xFAD mice. Kruskal-Wallis test followed by Dunn’s multiple comparison test, *P = 0.03, *P = 0.03. **d** Fluorescence microscopy of Aβ (red) and DAPI (blue). Shown are representative images of the cortex from female 5xFAD mice either non pregnant, pregnant but not lactating or pregnant and lactating at P20. **e** Quantification of Aβ plaque load in the cortex exhibited significant increases in pregnant and lactating 5xFAD mice compared to nulliparous 5xFAD female mice. Kruskal-Wallis test followed by Dunn’s multiple comparison test, *p = 0.04, *p = 0.03. **f** Fluorescence microscopy of Aβ (red) and DAPI (blue). Shown are representative images of the olfactory bulb from female 5xFAD mice either non-pregnant, pregnant but not lactating or pregnant and lactating at P20. **g** Analysis of the number of compact Aβ plaques in the olfactory bulb exhibited significant increases in pregnant and lactating 5xFAD mice compared to nulliparous 5xFAD female mice. Kruskal-Wallis test followed by Dunn’s multiple comparison test, *P* = 0.056, *P = 0.02. Data are presented as mean ± S.D. Each symbol represents data from one mouse, with four to seven mice per group. Scale bar represents 500 μm. P = postpartum day

In search of a more detailed approach towards the relationship between pregnancy, motherhood and Aβ deposition, we tested the hypothesis that lactation itself might have an effect on Aβ pathology. Therefore, we mated 12 weeks old 5xFAD mice and subdivided the females after birth into the following 3 groups: a) non-pregnant, b) pregnant but non-lactating and c) pregnant and lactating. All mice were analyzed 4 weeks after delivery at postpartum day 20 (P20) (Fig. 4a). Aβ immunohistochemistry revealed a significantly higher Aβ plaque load in pregnant and lactating female mice compared to non-pregnant mice. Female mice that delivered but were separated from their litter directly after birth had enhanced hippocampal Aβ plaque numbers compared to non-pregnant ones but did not differ from the lactating group (Fig. 4b and c). To confirm these observations in other brain regions, Aβ plaques were analyzed in the cortex and olfactory bulb of the same 5xFAD mice by immunohistochemistry (Fig. 4d and f). In accordance with the results obtained in the hippocampus, the total and compact Aβ plaque load was significantly enhanced in pregnant and lactating female 5xFAD mice at P20 (Fig. 4e), albeit not reaching significance for the total Aβ plaque load in the olfactory bulb (Fig. 4g). In summary, we conclude from these results that pregnancy alone is sufficient to induce this phenotype as lactation has no influence on this pathology.

### Adult hippocampal neurogenesis and cell proliferation are decreased during pregnancy and lactation period in 5xFAD mice

**Fig. 5:**
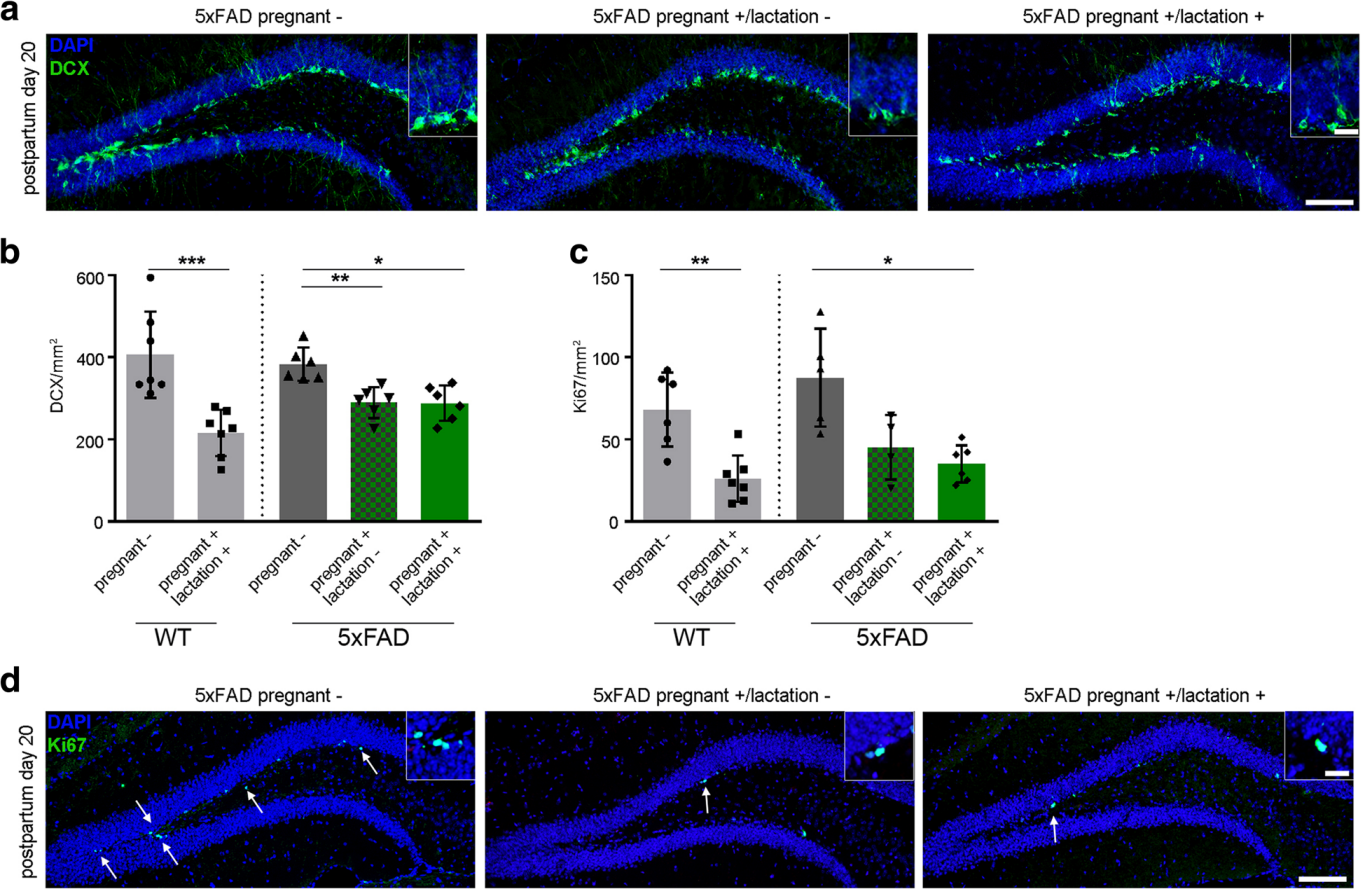
Decreased adult neurogenesis in pregnant and lactating WT and 5xFAD female mice at P20. **a** Fluorescence microscopy of DCX (green) and DAPI (blue). Shown are representative images of the dentate gyrus from 5xFAD female mice sacrificed at the postpartum day 20 (P20). Inserts show higher magnifications. **b** Quantification of DCX positive cells displayed significant reductions in pregnant and lactating WT and 5xFAD female mice at P20. Mann-Whitney test, **P = 0.0015, Kruskal-Wallis test followed by Dunn’s multiple comparison test, **P = 0.0089, *P = 0.012. **c** Quantification of Ki67 positive cells exhibited significant reductions in pregnant and lactating WT and 5xFAD female mice at P20. Mann-Whitney test, **P = 0.0047, Kruskal-Wallis test followed by Dunn’s multiple comparison test, *P = 0.02. **d** Fluorescence microscopy of Ki67 (green) and DAPI (blue). Shown are representative images of the dentate gyrus from 5xFAD female mice sacrificed at P20. White arrows indicate the presence of Ki67 positive cells. Inserts show higher magnifications. Scale bar represents 100 μm in the overview and 20 μm in the inserts. Data are presented as mean ± S.D. Each symbol represents data from one mouse, with five to seven mice per group. P = postpartum day, DCX = doublecortin

We determined proliferation and neurogenesis in 5xFAD transgenic and WT mice at P20 and compared them to their non-pregnant littermates to study the effect of pregnancy, lactation and Aβ plaque pathology on cells. While the number of doublecortin (DCX) positive cells dramatically declined during pregnancy in WT and 5xFAD mice, the number of DCX positive neuroblasts and immature neurons did not differ between pregnant/non-lactating and pregnant/lactating 5xFAD females at P20 (Fig. 5a and b), corroborating the aforementioned effect of pregnancy alone. Likewise, the number of Ki67 positive proliferating cells in pregnant WT and 5xFAD mice was significantly decreased (Fig. 5c and d).

### Exposure to environmental enrichment and voluntary running during pregnancy circumvents Aβ plaque pathology and vivifies neurogenesis

**Fig. 6:**
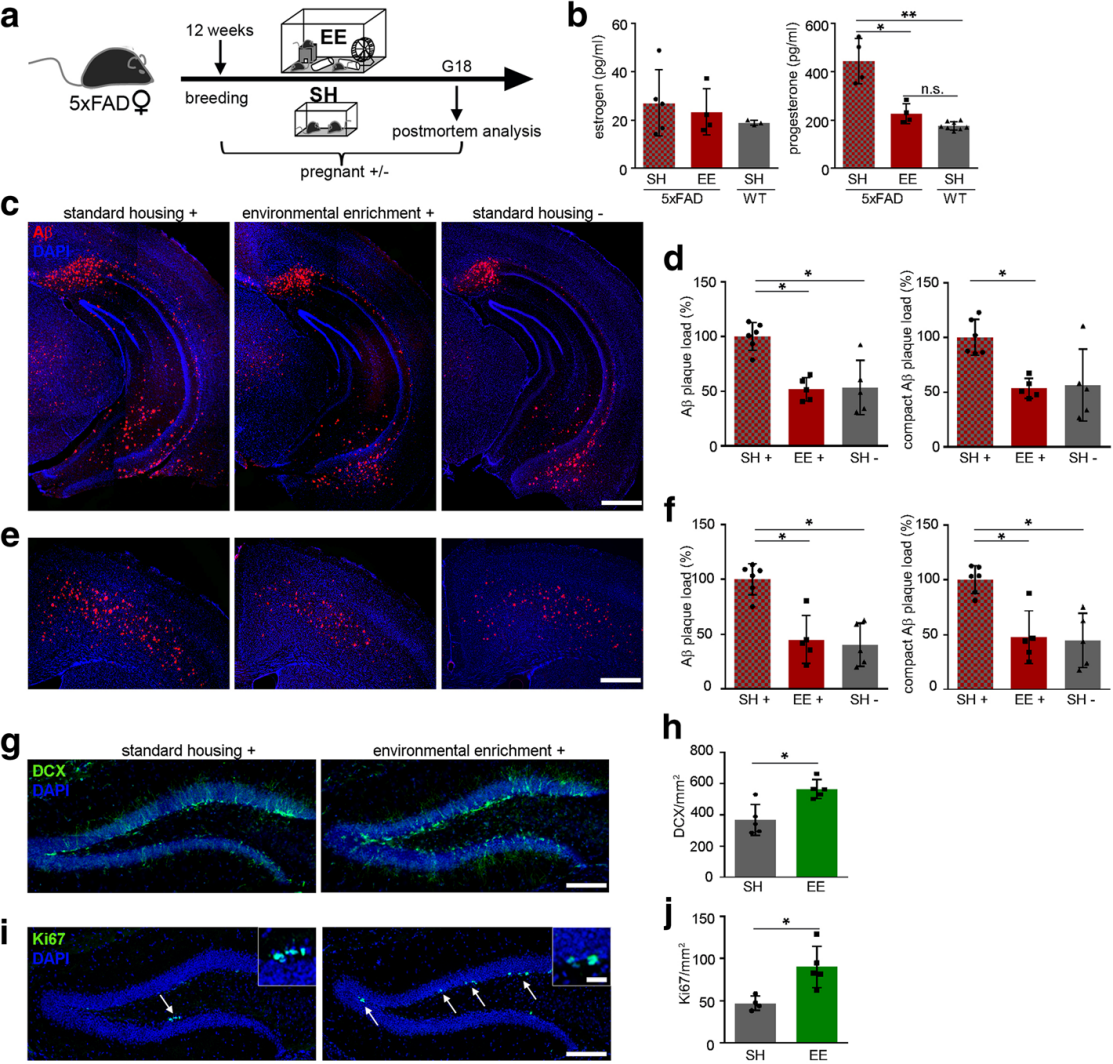
Environmental enrichment prevents increase in Aβ plaque pathology during pregnancy in 5xFAD mice. **a** Schematic overview of the experimental setup. Twelve weeks old 5xFAD female mice were mated and either housed in standard housing (SH) or in environmental enrichment (EE) until they were sacrificed at gestation day 18 (G18). **b** Serum estrogen and progesterone levels of pregnant 5xFAD and WT female mice exposed to EE or SH were measured by ELISA at G18. Estrogen levels did not differ between EE and SH housed female mice. Progesterone levels decreased in 5xFAD mice housed in EE to the level of standard housed pregnant WT mice. Kruskal-Wallis test followed by Dunn’s multiple comparison test, *P = 0.03, **P = 0.0025. **c** Fluorescence microscopy of Aβ (red) and DAPI (blue). Shown are representative images of hippocampi from pregnant female 5xFAD mice housed in SH or EE and non-pregnant housed in SH. **d** Quantification of Aβ plaque load (left) and compact Aβ plaque load (right) of pregnant 5xFAD mice housed in SH or EE displayed significant decreases in the number of Aβ plaques after housing in EE. Kruskal-Wallis test followed by Dunn’s multiple comparison test, *P = 0.027, *P = 0.0178, *P = 0.0364. **e** Fluorescence microscopy of Aβ (red) and DAPI (blue). Shown are representative images of the cortex from pregnant female 5xFAD mice housed in SH or EE and non-pregnant mice housed in SH. **f** Quantification of Aβ plaque load in the cortex of pregnant 5xFAD mice at G18 exhibited significant decreases in mice housed in EE. Kruskal-Wallis test followed by Dunn’s multiple comparison test, *P = 0.040, *P = 0.012, *P = 0.033, *P = 0.014. **g** Fluorescence microscopy of DCX (green) and DAPI (blue). Shown are representative images of the dentate gyrus from pregnant 5xFAD female mice housed in SH or EE sacrificed at G18. **h** Quantification of DCX positive cells in pregnant 5xFAD mice showed significant increases in mice housed in EE. Mann-Whitney test, *P = 0.03. **i** Fluorescence microscopy of Ki67 (green) and DAPI (blue). Shown are representative images of the dentate gyrus from pregnant female 5xFAD mice housed in SH or EE. **j** Analysis of Ki67 positive cells in pregnant 5xFAD mice revealed significant increases in mice housed in EE. Mann-Whitney test, *P = 0.02. Inserts show higher magnifications. White arrows indicate Ki67 positive cells. Scale bar represents 500 μm in (**c** and **e**) and 100 μm in (g and i) and 20 μm in the insert. Data are presented as mean ± S.D. Each symbol represents data from one mouse, with four to six mice per group. SH = standard housing, EE = environmental enrichment, DCX = doublecortin, G = gestation day

Inspired by studies that report beneficial effects on the Aβ plaque burden in APP transgenic mice [22] upon exposure to an environmental enrichment (EE) and voluntary running, we exposed pregnant 5xFAD mice to EE during 18 days, based on a published protocol [3], and compared them with pregnant 5xFAD mice housed in standard cages (SH) (Fig. 6a). First, we focused on the question if exposure of the mice to an enriched environment influences their estrogen and progesterone levels. Indeed, progesterone levels of pregnant 5xFAD mice housed in EE dropped significantly to levels that were comparable to WT pregnant mice housed in SH, while estrogen levels remained unaffected (Fig. 6b). Intriguingly, hippocampal and cortical Aβ plaque pathology was significantly decreased in pregnant 5xFAD mice housed in EE compared to pregnant mice housed in SH and even reached an Aβ plaque load similar to the non-pregnant control group housed in SH (Fig. 6c – f).

Since earlier studies reported beneficial effects on adult hippocampal neurogenesis upon exposure to an environmental enrichment (EE) [18, 19] and voluntary running [37, 38], we focused on the question whether EE could be beneficial for pregnant 5xFAD mice as well, by stimulating cell proliferation and adult neurogenesis. Consistent with the literature [18], pregnant 5xFAD mice housed under SH conditions had significantly less DCX labeled cells compared to mice housed in EE (Fig. 6g and h). Moreover, by quantifying Ki67 positive cells, it was clearly evident that pregnant mice housed in EE had significantly more proliferation compared to their counterparts in SH (Fig. 6i and j).

**Fig. 7:**
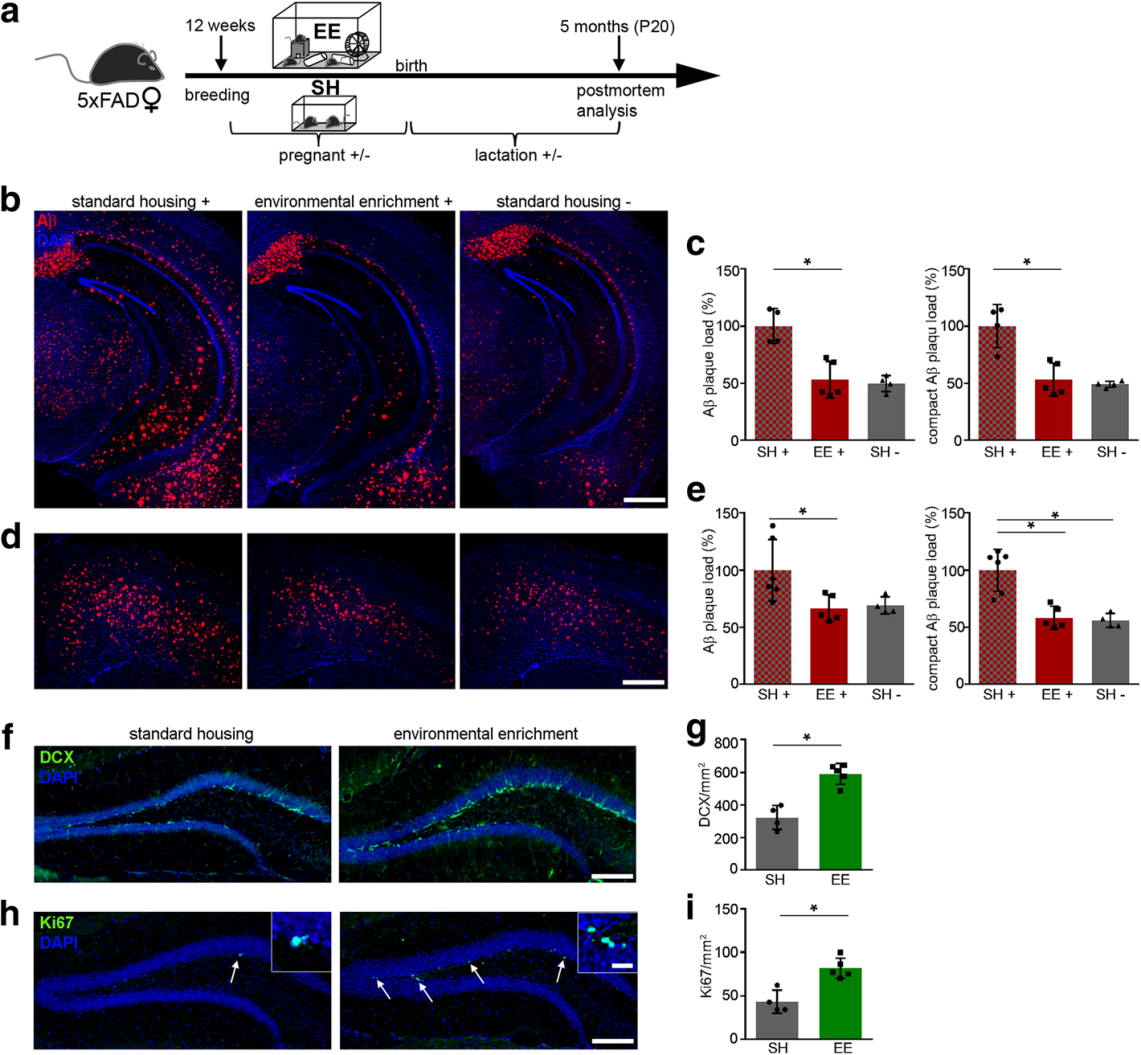
Environmental enrichment during pregnancy and lactation rescues Aβ plaque pathology in 5xFAD mice. **a** Schematic overview of the experimental setup. Twelve weeks old 5xFAD female mice were mated and either housed in standard housing (SH) or in environmental enrichment (EE) until they were sacrificed at postpartum day 20 (P20). **b** Fluorescence microscopy of Aβ (red) and DAPI (blue). Shown are representative images of hippocampi from pregnant/lactating female 5xFAD mice housed in SH or EE and non-pregnant females housed in SH. **c** Quantification of Aβ plaque load (left) and compact Aβ plaque load (right) of pregnant/lactating 5xFAD mice housed in SH or EE displayed significant decreases in the number of Aβ plaques after housing in EE. Kruskal-Wallis test followed by Dunn’s multiple comparison test, *P = 0.039, *P = 0.03. **d** Fluorescence microscopy of Aβ (red) and DAPI (blue). Shown are representative images of the cortex from pregnant/lactating female 5xFAD mice at P20 housed in SH or EE and non-pregnant females housed in SH. **e** Quantification of Aβ plaque load in the cortex of pregnant/lactating 5xFAD mice at P20 exhibited significant decreases in mice housed in EE. Kruskal-Wallis test followed by Dunn’s multiple comparison test, *P = 0.024, *P = 0.017. **f** Fluorescence microscopy of DCX (green) and DAPI (blue). Shown are representative images of the dentate gyrus from pregnant 5xFAD female mice housed in SH or EE sacrificed at P20. **g** Quantification of DCX positive cells in pregnant/lactating 5xFAD mice showed significant increases in mice housed in EE. Mann-Whitney test, *P = 0.015. **h** Fluorescence microscopy of Ki67 (green) and DAPI (blue). Shown are representative images of the dentate gyrus from pregnant female 5xFAD mice housed in SH or EE. **i** Analysis of Ki67 positive cells in pregnant/lactating 5xFAD mice revealed significant increases in mice housed in EE. Mann-Whitney test, *P = 0.015. Inserts show higher magnifications. White arrows indicate Ki67 positive cells. Scale bar represents 500 μm in (**b** and **d**) and 100 μm in (**f** and **h**) and 20 μm in the insert. Data are presented as mean ± S.D. Each symbol represents data from one mouse, with four to six mice per group. SH = standard housing, EE = environmental enrichment, DCX = doublecortin, P = postpartum day

Given the positive effects of EE during pregnancy, we then tested whether the increased Aβ plaque burden during pregnancy and lactation could be reduced or even halted by exposure to an EE. Therefore, 5xFAD mice were mated at the age of 12 weeks and exposed directly to EE from G1 onwards until the end of the weaning period (P20) (Fig. 7a). Indeed, quantitative analysis of Aβ plaque numbers displayed a reduced hippocampal plaque burden in lactating mice housed in EE compared to mice housed in SH that even were comparable to those of the non-pregnant control group housed in SH (Fig. 7b and c). Similar results were obtained by examining Aβ plaques in the cortex (Fig. 7d and e), suggesting an overall beneficial effect of EE on Aβ plaque pathology.

Finally, we studied the influence of EE on cell proliferation and adult neurogenesis in pregnant and lactating 5xFAD mice. Consistent with our previous findings, housing 5xFAD female mice during pregnancy and the lactation period (P20) in EE resulted in a significantly enhanced number of DCX and Ki67 labeled cells compared to mice housed in SH (Fig. 7f – i), thus clearly indicating the beneficial effect of EE during pregnancy.

## Discussion

Age and gender are the greatest non-genetic risk factors for developing sporadic AD. There are indications that reproductive experiences are the cause for an earlier onset of AD in women. However, only few studies have investigated the underlying mechanism for the higher risk of AD in women [6, 7, 31, 33]. Furthermore, the impact of pregnancy on Aβ plaque pathology and adult neurogenesis still remains elusive. To this end, we analyzed pregnant 5xFAD transgenic mice at different gestation days by immunohistochemical stainings and determined the Aβ plaque load as well as proliferation and neurogenesis. Indeed we found that pregnancy alters Aβ plaque pathology in female 5xFAD mice and reduces cell proliferation and the generation of newborn neurons in the hippocampus. Considering that female 5xFAD transgenic mice that also nursed their offspring for 4 weeks had a similar Aβ plaque load and Ki67- and DCX cell counts as merely pregnant mice, we conclude that pregnancy alone is sufficient to exacerbate this pathology.

Epidemiological studies reported that women with multiple pregnancies have an earlier onset of AD and that nulliparous women show less age-related cognitive decline [6, 7, 25, 31]. Our data do not support this view since we could not detect any difference in Aβ plaque load between nulliparous and multiparous 5xFAD female mice, suggesting no higher risk for multiparous female mice. However, we can’t exclude the possibility that a significant difference would have been detectable in a slower Aβ aggregation mouse model such as APP23 or Tg2576 with an overall lower Aβ plaque burden at that age. Future studies will need to address this question. In further support of a mechanistic link between pregnancy, motherhood and an increased risk for the development of AD, it is observed that nulliparous women have higher estrogen levels later in life compared to women with reproductive experiences [2, 23]. Nevertheless, it is still under debate if estrogens could also have protective functions and eventually delay the onset of AD [11, 24, 28]. However, most findings in human studies should be taken cautiously because they contain insufficient information on the usage of birth control pills, especially in nulliparous women. While our results demonstrate a substantial increase in Aβ plaque formation during the course of pregnancy in 5xFAD transgenic mice compared to non-pregnant littermates, they also suggest that progesterone rather than estrogen levels might account for this result since estrogen levels were similar in pregnant WT and 5xFAD transgenic mice. The question whether estrogen levels change during aging in 5xFAD transgenic mice still remains open. This could give additional interesting hints specifically in comparison to the situation in humans. Recent work in rats has demonstrated that pregnancy-related hormones might also play a role in Aβ clearance by influencing Aβ clearance factors such as IDE [17]. In agreement with the proposed relationship between levels of pregnancy-related hormones and IDE [17], we indeed found higher IDE levels in pregnant than in non-pregnant 5xFAD mice that also had high progesterone levels. It still remains an open question why pregnant 5xFAD mice had increased Aβ levels despite elevated IDE levels that most likely were not able to execute its Aβ-degrading function. In contrast, neprilysin levels were not altered. Together, these experiments suggest that both, IDE and neprilysin did not affect the clearance of Aβ in a way that could account for the increased plaque density in pregnant mice. Instead, the high amount of CTF-β in pregnant mice might rather indicate that the observed increase in amyloid plaque density was primarily due to alterations in APP processing and Aβ production.

Given that lactation might also affect pathology, we further included non-lactating and lactating 5xFAD mice in our study, in order to determine if lactation could enlarge pathological changes during the postpartum period. In agreement with other studies we were able to confirm that breeders display higher numbers of Aβ plaques compared to non-breeders [8]. Importantly, this finding was applicable to other brain regions, such as the cortex and the olfactory bulb. Interestingly, there was no difference in Aβ plaque pathology between 5xFAD mice that nursed their offspring and those separated from the offspring directly after birth. Therefore we concluded that pregnancy alone is sufficient to induce this phenotype.

Reproductive experience, pregnancy and lactation influence physiological and endocrinological systems in women [4, 30, 32, 36]. Reproductive hormones that are produced during pregnancy and the postpartum period are known to alter neurogenesis and dendritic morphology [30, 32]. Cognitive deficits after reproductive experiences have been reported in human as well as in mouse studies [8, 12], although with inconclusive and contradictory results. While Cui et al [8] were able to show that WT breeders outperformed non-breeders in spatial working and memory they found the opposite in APP transgenic mice. Several studies provided evidence for the involvement of hippocampal neurogenesis in learning and memory and for its alteration under disease conditions such as AD and during reproductive experiences [10, 15, 20, 21]. To establish whether adult hippocampal neurogenesis is altered during the course of pregnancy and in the postpartum period, we carried out a detailed analysis and quantified Ki67 and DCX-positive cells. Indeed, adult hippocampal neurogenesis and cell proliferation were significantly diminished in 5 month old pregnant and lactating 5xFAD mice, suggesting that in the course of pregnancy the generation and maturation of newborn neurons is disturbed. Again, the lactation period alone had no influence on cell proliferation and adult neurogenesis. In summary, our data is in support of several human studies claiming that pregnancy is a risk factor for AD.

Previous work has demonstrated that voluntary running and EE can have beneficial effects on Aβ plaque formation and adult hippocampal neurogenesis [16, 18, 19, 22, 26, 37, 38, 41]. While there is no data published regarding the impact of voluntary running and EE on pregnant and lactating mothers, it has been reported that running during pregnancy reduces the Aβ plaque burden in transgenic offspring [13]. Inspired by a study that reports high running activity of pregnant mice until gestation day 10 [3], we exposed pregnant mice to an EE and investigated Aβ plaque pathology and adult neurogenesis during pregnancy and in the postpartum period. Intriguingly, maternal voluntary running and exercise prevented the pregnancy related increase in Aβ plaque pathology and stimulated cell proliferation as well as the generation of newborn neurons during the course of pregnancy. This beneficial effect of EE during the course of pregnancy was even more pronounced after the lactation period at P20, suggesting stronger effects with longterm EE. It remains to be seen if housing the pregnant mice in EE provides protection early on e.g. already at G10. Future experiments will have to test whether maternal voluntary running and exercise have long lasting effects on Aβ plaque pathology and adult neurogenesis as suggested by others [3] and whether maternal activity might lead to changes in hormone levels, such as estrogens or progesterone.

## Conclusion

In conclusion, the present study demonstrates that Aβ plaque formation, cell proliferation and adult neurogenesis are dynamic processes, which can be influenced by reproductive activity on the one hand and physical exercise on the other hand. Together, our findings are unique and underscore the vulnerability of female brains during pregnancy and the effectiveness of EE as a non-pharmacological approach to counteract this happening.

## References

[1] Bayer TA, Schafer S, Simons A, Kemmling A, Kamer T, Tepest R, Eckert A, Schussel K, Eikenberg O, Sturchler-Pierrat C (2003). Dietary cu stabilizes brain superoxide dismutase 1 activity and reduces amyloid Abeta production in APP23 transgenic mice. Proc Natl Acad Sci U S A.

[2] Bernstein L, Pike MC, Ross RK, Judd HL, Brown JB, Henderson BE (1985). Estrogen and sex hormone-binding globulin levels in nulliparous and parous women. J Natl Cancer Inst.

[3] Bick-Sander A, Steiner B, Wolf SA, Babu H, Kempermann G (2006). Running in pregnancy transiently increases postnatal hippocampal neurogenesis in the offspring. Proc Natl Acad Sci U S A.

[4] Bridges RS (2016). Long-term alterations in neural and endocrine processes induced by motherhood in mammals. Horm Behav.

[5] Callahan MJ, Lipinski WJ, Bian F, Durham RA, Pack A, Walker LC (2001). Augmented senile plaque load in aged female beta-amyloid precursor protein-transgenic mice. Am J Pathol.

[6] Colucci M, Cammarata S, Assini A, Croce R, Clerici F, Novello C, Mazzella L, Dagnino N, Mariani C, Tanganelli P (2006). The number of pregnancies is a risk factor for Alzheimer's disease. Eur J Neurol.

[7] Corbo RM, Gambina G, Ulizzi L, Monini P, Broggio E, Rosano A, Scacchi R (2007). Combined effect of apolipoprotein e genotype and past fertility on age at onset of Alzheimer's disease in women. Dement Geriatr Cogn Disord.

[8] Cui J, Jothishankar B, He P, Staufenbiel M, Shen Y, Li R (2014). Amyloid precursor protein mutation disrupts reproductive experience-enhanced normal cognitive development in a mouse model of Alzheimer's disease. Mol Neurobiol.

[9] Cummings JL (2004). Alzheimer's disease. N Engl J Med.

[10] Ekonomou A, Savva GM, Brayne C, Forster G, Francis PT, Johnson M, Perry EK, Attems J, Somani A, Minger SL (2015). Stage-specific changes in neurogenic and glial markers in Alzheimer's disease. Biol Psychiatry.

[11] Henderson VW, Paganini-Hill A, Emanuel CK, Dunn ME, Buckwalter JG (1994). Estrogen replacement therapy in older women. Comparisons between Alzheimer's disease cases and nondemented control subjects. Arch Neurol.

[12] Henry JF, Sherwin BB (2012). Hormones and cognitive functioning during late pregnancy and postpartum: a longitudinal study. Behav Neurosci.

[13] Herring A, Donath A, Yarmolenko M, Uslar E, Conzen C, Kanakis D, Bosma C, Worm K, Paulus W, Keyvani K (2012). Exercise during pregnancy mitigates Alzheimer-like pathology in mouse offspring. FASEB J.

[14] Hirata-Fukae C, Li HF, Hoe HS, Gray AJ, Minami SS, Hamada K, Niikura T, Hua F, Tsukagoshi-Nagai H, Horikoshi-Sakuraba Y (2008). Females exhibit more extensive amyloid, but not tau, pathology in an Alzheimer transgenic model. Brain Res.

[15] Hollands C, Bartolotti N, Lazarov O (2016). Alzheimer's disease and hippocampal adult neurogenesis; exploring shared mechanisms. Front Neurosci.

[16] Hu YS, Xu P, Pigino G, Brady ST, Larson J, Lazarov O (2010). Complex environment experience rescues impaired neurogenesis, enhances synaptic plasticity, and attenuates neuropathology in familial Alzheimer's disease-linked APPswe/PS1DeltaE9 mice. FASEB J.

[17] Jayaraman A, Carroll JC, Morgan TE, Lin S, Zhao L, Arimoto JM, Murphy MP, Beckett TL, Finch CE, Brinton RD (2012). 17beta-estradiol and progesterone regulate expression of beta-amyloid clearance factors in primary neuron cultures and female rat brain. Endocrinology.

[18] Kempermann G, Kuhn HG, Gage FH (1997). More hippocampal neurons in adult mice living in an enriched environment. Nature.

[19] Kempermann G, Kuhn HG, Gage FH (1998). Experience-induced neurogenesis in the senescent dentate gyrus. J Neurosci.

[20] Lazarov O, Marr RA (2010). Neurogenesis and Alzheimer's disease: at the crossroads. Exp Neurol.

[21] Lazarov O, Mattson MP, Peterson DA, Pimplikar SW, van Praag H (2010). When neurogenesis encounters aging and disease. Trends Neurosci.

[22] Lazarov O, Robinson J, Tang YP, Hairston IS, Korade-Mirnics Z, Lee VM, Hersh LB, Sapolsky RM, Mirnics K, Sisodia SS (2005). Environmental enrichment reduces Abeta levels and amyloid deposition in transgenic mice. Cell.

[23] Lee JH, Jiang Y, Han DH, Shin SK, Choi WH, Lee MJ (2014). Targeting estrogen receptors for the treatment of Alzheimer's disease. Mol Neurobiol.

[24] Liang K, Yang L, Yin C, Xiao Z, Zhang J, Liu Y, Huang J (2010). Estrogen stimulates degradation of beta-amyloid peptide by up-regulating neprilysin. J Biol Chem.

[25] McLay RN, Maki PM, Lyketsos CG (2003). Nulliparity and late menopause are associated with decreased cognitive decline. J Neuropsychiatry Clin Neurosci.

[26] Nilsson M, Perfilieva E, Johansson U, Orwar O, Eriksson PS (1999). Enriched environment increases neurogenesis in the adult rat dentate gyrus and improves spatial memory. J Neurobiol.

[27] Oakley H, Cole SL, Logan S, Maus E, Shao P, Craft J, Guillozet-Bongaarts A, Ohno M, Disterhoft J, Van Eldik L (2006). Intraneuronal beta-amyloid aggregates, neurodegeneration, and neuron loss in transgenic mice with five familial Alzheimer's disease mutations: potential factors in amyloid plaque formation. J Neurosci.

[28] Paganini-Hill A, Henderson VW (1994). Estrogen deficiency and risk of Alzheimer's disease in women. Am J Epidemiol.

[29] Paxinos G, Franklin KBJ (2001) The mouse brain in stereotaxic coordinates 2n edn. San Diego, Academic Press

[30] Prange-Kiel J, Rune GM (2006). Direct and indirect effects of estrogen on rat hippocampus. Neuroscience.

[31] Ptok U, Barkow K, Heun R (2002). Fertility and number of children in patients with Alzheimer's disease. Arch Womens Ment Health.

[32] Shingo T, Gregg C, Enwere E, Fujikawa H, Hassam R, Geary C, Cross JC, Weiss S (2003). Pregnancy-stimulated neurogenesis in the adult female forebrain mediated by prolactin. Science.

[33] Sobow T, Kloszewska I (2004). Parity, number of pregnancies, and the age of onset of Alzheimer's disease. J Neuropsychiatry Clin Neurosci..

[34] Steiner H, Kostka M, Romig H, Basset G, Pesold B, Hardy J, Capell A, Meyn L, Grim ML, Baumeister R (2000). Glycine 384 is required for presenilin-1 function and is conserved in bacterial polytopic aspartyl proteases. Nat Cell Biol.

[35] Sturchler-Pierrat C, Staufenbiel M (2000). Pathogenic mechanisms of Alzheimer's disease analyzed in the APP23 transgenic mouse model. Ann N Y Acad Sci.

[36] Tomizawa K, Iga N, Lu YF, Moriwaki A, Matsushita M, Li ST, Miyamoto O, Itano T, Matsui H (2003). Oxytocin improves long-lasting spatial memory during motherhood through MAP kinase cascade. Nat Neurosci.

[37] van Praag H, Christie BR, Sejnowski TJ, Gage FH (1999). Running enhances neurogenesis, learning, and long-term potentiation in mice. Proc Natl Acad Sci U S A.

[38] van Praag H, Kempermann G, Gage FH (1999). Running increases cell proliferation and neurogenesis in the adult mouse dentate gyrus. Nat Neurosci.

[39] Wang J, Tanila H, Puolivali J, Kadish I, van Groen T (2003). Gender differences in the amount and deposition of amyloidbeta in APPswe and PS1 double transgenic mice. Neurobiol Dis.

[40] Yamasaki A, Eimer S, Okochi M, Smialowska A, Kaether C, Baumeister R, Haass C, Steiner H (2006). The GxGD motif of presenilin contributes to catalytic function and substrate identification of gamma-secretase. J Neurosci.

[41] Ziegler-Waldkirch S, d'Errico P, Sauer JF, Erny D, Savanthrapadian S, Loreth D, Katzmarski N, Blank T, Bartos M, Prinz M (2018). Seed-induced Abeta deposition is modulated by microglia under environmental enrichment in a mouse model of Alzheimer’s disease. EMBO J.

